# Bis(μ-3,5-difluorobenzoato)bis[(3,5-di­fluorobenzoato)dimethyltin(IV)]

**DOI:** 10.1107/S1600536810054061

**Published:** 2011-01-08

**Authors:** Hong Liu, Han-Dong Yin, Jing Li, Da-Qi Wang

**Affiliations:** a College of Chemistry and Chemical Engineering, Liaocheng University, Shandong 252059, People’s Republic of China

## Abstract

In the dinuclear title complex, [Sn_2_(CH_3_)_4_(C_7_H_3_F_2_O_2_)_4_], the Sn^IV^ atom is chelated by two 3,5-difluoro­benzoate (dfb) anions and coordinated by two methyl groups while an O atom from the adjacent dfb anion bridges the Sb atom with a longer Sb—O bond distance of 2.793 (4) Å. The complex mol­ecule has 2 symmetry and the Sn^IV^ atom is in a distorted penta­gonal–bipyramidal coordination geometry. In the crystal, mol­ecules are connected by C—H⋯O and C—H⋯F hydrogen bonds.

## Related literature

For applications of organotin compounds, see: Duboy & Roy (2003 ▶). For related compounds, see: Yin *et al.* (2003 ▶, 2005 ▶).
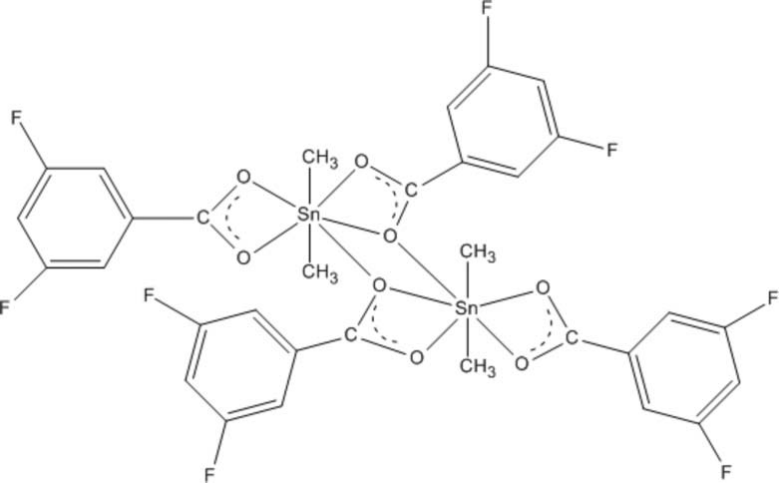


## Experimental

### 

#### Crystal data

[Sn_2_(CH_3_)_4_(C_7_H_3_F_2_O_2_)_4_]
*M* *_r_* = 925.90
Monoclinic, 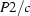

*a* = 16.4635 (15) Å
*b* = 7.5836 (8) Å
*c* = 15.1123 (14) Å
β = 115.680 (1)°
*V* = 1700.4 (3) Å^3^
*Z* = 2
Mo *K*α radiation
μ = 1.56 mm^−1^
*T* = 298 K
0.49 × 0.43 × 0.18 mm

#### Data collection

Bruker SMART CCD area detector diffractometer
Absorption correction: multi-scan (*SADABS*; Sheldrick, 1996 ▶) *T*_min_ = 0.515, *T*_max_ = 0.766
8140 measured reflections
2987 independent reflections
2020 reflections with *I* > 2σ(*I*)
*R*_int_ = 0.059

#### Refinement

*R*[*F* ^2^ > 2σ(*F* ^2^)] = 0.043
*wR*(*F* ^2^) = 0.127
*S* = 1.05
2987 reflections
228 parameters
H-atom parameters constrained
Δρ_max_ = 1.47 e Å^−3^
Δρ_min_ = −0.76 e Å^−3^

### 

Data collection: *SMART* (Siemens, 1996 ▶); cell refinement: *SAINT* (Siemens, 1996 ▶); data reduction: *SAINT*; program(s) used to solve structure: *SHELXTL* (Sheldrick, 2008 ▶); program(s) used to refine structure: *SHELXTL*; molecular graphics: *SHELXTL*; software used to prepare material for publication: *SHELXTL*.

## Supplementary Material

Crystal structure: contains datablocks I, global. DOI: 10.1107/S1600536810054061/xu5108sup1.cif

Structure factors: contains datablocks I. DOI: 10.1107/S1600536810054061/xu5108Isup2.hkl

Additional supplementary materials:  crystallographic information; 3D view; checkCIF report

## Figures and Tables

**Table 1:** Selected bond lengths (Å)

Sn1—O1	2.534 (4)
Sn1—O2	2.163 (4)
Sn1—O3	2.424 (4)
Sn1—O4	2.155 (4)
Sn1—C15	2.093 (6)
Sn1—C16	2.088 (6)

**Table 2:** Hydrogen-bond geometry (Å, °)

*D*—H⋯*A*	*D*—H	H⋯*A*	*D*⋯*A*	*D*—H⋯*A*
C11—H11⋯F4^i^	0.93	2.49	3.326 (8)	149
C15—H15*B*⋯O4^ii^	0.96	2.52	3.474 (8)	171

Symmetry codes: (i) 
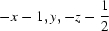
; (ii) 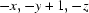.

**Fig. 1.**
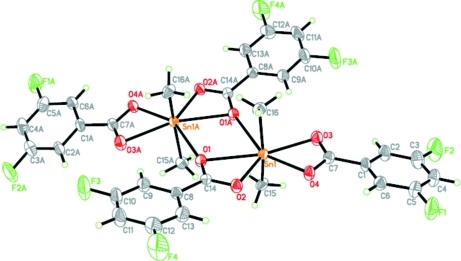
The molecular structure of the compound, showing 50% probability displacement ellipsoids [symmetry code: (A) -x, y, -z + 1/2].

## supplementary crystallographic information

### Comment

In recent years, organotin compounds have attracted increasing attention owing to their wide industrial applications and biological activities (Duboy & Roy, 2003). We have therefore synthesized the title compound, and present its crystal structure here. The molecular structure of the compound is shown Fig. 1. For this compound, the asymmetric unit contains twomonomers, which are different from a crystallographic point of view. In this compound we can found the Sn atom exists in a distorted pentagonal bipyramidal coordination environment. The atoms O1, O1A, O2, O3 and O4 are coplanar within 0.044 Å, which form the equatorial plane. Furthermore, the angle of the axial C16—Sn1—C15 is 156.8 (3)°, which deviates from the linear angle of 180. The O1 atom of the carboxylate residue also binds the other tin atom, Sn1A, generating a Sn2O2 four-membered ring. The distance of Sn—O1 [-*x*, *y*, 0.5 - *z*] (2.793 (4) Å) is relatively longer than that of Sn1—O1 (2.534 (4) Å), but is comparable to those found in related seven-coordinate diorganotin systems (Yin *et al.*, 2003). Thereby, the molecular structure of this compound can be described as a dimer, and the coordination geometry of tin can also be described as a *trans*-C_2_SnO_5_ pentagonal bipyramid with the two methyl groups occupying axial positions (Yin *et al.*, 2005).

The molecules are linked by C—H···O and C—H···F hydrogen bonds into a one-dimensional chain structure (Table 2).

### Experimental

3,5-Difluorobenzoic acid (0.4 mmol) was added to a methanol solution of sodium ethoxide (0.4 mmol) and heated at reflux for 0.5 h. To this solution was added dimethyltin dichloride (0.2 mmol) in benzene, and the mixture was refluxed for 5 h, cooled and filtered. The filtrate was evaporated *in vacuo*. The obtained solid was recrystallized from dichloromethane-petroleum ether. Anal. Calcd (%) for C_16_H_12_F_4_O_4_Sn (Mr = 462.95): C, 41.48; H, 2.60; Found (%): C, 41.49; H, 2.61.

### Refinement

The H atoms were positioned geometrically, with C—H = 0.96 (emthyl) and C—H = 0.93 Å (aromatic), and refined as riding on parent atoms with *U*_iso_(H) = 1.5*U*_eq_(C) for methyl and 1.2*U*_eq_(C) for the others.

### Crystal data

**Table:**

[Sn_2_(C_7_H_3_F_2_O_2_)_4_(CH_3_)_4_]	*F*(000) = 904
*M**_r_* = 925.90	*D*_x_ = 1.808 Mg m^−^^3^
Monoclinic, *P*2/*c*	Mo *K*α radiation, λ = 0.71073 Å
Hall symbol: -P 2yc	Cell parameters from 2614 reflections
*a* = 16.4635 (15) Å	θ = 2.7–26.6°
*b* = 7.5836 (8) Å	µ = 1.56 mm^−^^1^
*c* = 15.1123 (14) Å	*T* = 298 K
β = 115.680 (1)°	Block, colourless
*V* = 1700.4 (3) Å^3^	0.49 × 0.43 × 0.18 mm
*Z* = 2	

### Data collection

**Table:**

Bruker SMART CCD area detector diffractometer	2987 independent reflections
Radiation source: fine-focus sealed tube	2020 reflections with *I* > 2σ(*I*)
graphite	*R*_int_ = 0.059
φ and ω scans	θ_max_ = 25.0°, θ_min_ = 2.7°
Absorption correction: multi-scan (*SADABS*; Sheldrick, 1996)	*h* = −19→18
*T*_min_ = 0.515, *T*_max_ = 0.766	*k* = −8→9
8140 measured reflections	*l* = −17→14

### Refinement

**Table:**

Refinement on *F*^2^	Primary atom site location: structure-invariant direct methods
Least-squares matrix: full	Secondary atom site location: difference Fourier map
*R*[*F*^2^ > 2σ(*F*^2^)] = 0.043	Hydrogen site location: inferred from neighbouring sites
*wR*(*F*^2^) = 0.127	H-atom parameters constrained
*S* = 1.05	*w* = 1/[σ^2^(*F*_o_^2^) + (0.0583*P*)^2^ + 0.6314*P*] where *P* = (*F*_o_^2^ + 2*F*_c_^2^)/3
2987 reflections	(Δ/σ)_max_ = 0.003
228 parameters	Δρ_max_ = 1.47 e Å^−^^3^
0 restraints	Δρ_min_ = −0.76 e Å^−^^3^

### Fractional atomic coordinates and isotropic or equivalent isotropic displacement parameters (Å^2^)

**Table:**

	*x*	*y*	*z*	*U*_iso_*/*U*_eq_	
Sn1	0.05503 (2)	0.25943 (4)	0.14161 (3)	0.03506 (18)	
O1	−0.0907 (3)	0.2541 (4)	0.1601 (3)	0.0409 (10)	
O2	−0.0783 (3)	0.2418 (4)	0.0214 (3)	0.0435 (10)	
O3	0.2139 (3)	0.2807 (6)	0.1809 (4)	0.0566 (12)	
C7	0.1896 (4)	0.2814 (7)	0.0903 (5)	0.0436 (15)	
C14	−0.1268 (4)	0.2370 (6)	0.0687 (5)	0.0355 (13)	
C8	−0.2252 (4)	0.2015 (7)	0.0135 (5)	0.0394 (14)	
C1	0.2555 (4)	0.3085 (8)	0.0476 (5)	0.0464 (16)	
O4	0.1056 (3)	0.2649 (5)	0.0319 (3)	0.0456 (11)	
C9	−0.2776 (4)	0.1879 (9)	0.0649 (5)	0.0507 (16)	
H9	−0.2528	0.2066	0.1324	0.061*	
C6	0.2292 (4)	0.2844 (8)	−0.0504 (5)	0.0499 (16)	
H6	0.1704	0.2521	−0.0917	0.060*	
C13	−0.2630 (4)	0.1759 (10)	−0.0862 (5)	0.0580 (17)	
H13	−0.2286	0.1869	−0.1211	0.070*	
C16	0.0715 (4)	−0.0095 (8)	0.1744 (5)	0.0531 (16)	
H16A	0.1342	−0.0398	0.1991	0.080*	
H16B	0.0374	−0.0763	0.1160	0.080*	
H16C	0.0507	−0.0359	0.2232	0.080*	
C2	0.3429 (4)	0.3579 (9)	0.1092 (5)	0.0579 (18)	
H2	0.3610	0.3744	0.1761	0.069*	
C10	−0.3662 (4)	0.1465 (10)	0.0133 (6)	0.0649 (19)	
C5	0.2920 (5)	0.3092 (10)	−0.0867 (6)	0.066 (2)	
C15	0.0618 (4)	0.5303 (7)	0.1706 (5)	0.0487 (16)	
H15A	0.0596	0.5497	0.2323	0.073*	
H15B	0.0117	0.5885	0.1193	0.073*	
H15C	0.1171	0.5768	0.1736	0.073*	
C11	−0.4055 (5)	0.1176 (11)	−0.0845 (6)	0.074 (2)	
H11	−0.4662	0.0879	−0.1176	0.088*	
C3	0.4014 (5)	0.3813 (10)	0.0687 (7)	0.075 (2)	
C4	0.3776 (5)	0.3597 (10)	−0.0286 (7)	0.075 (2)	
H4	0.4188	0.3790	−0.0545	0.090*	
C12	−0.3522 (5)	0.1341 (11)	−0.1328 (5)	0.072 (2)	
F1	0.2678 (4)	0.2848 (7)	−0.1838 (4)	0.1003 (17)	
F3	−0.4177 (3)	0.1298 (8)	0.0627 (4)	0.1096 (18)	
F4	−0.3896 (3)	0.1015 (9)	−0.2298 (3)	0.131 (2)	
F2	0.4862 (3)	0.4353 (7)	0.1280 (4)	0.1166 (19)	

### Atomic displacement parameters (Å^2^)

**Table:**

	*U*^11^	*U*^22^	*U*^33^	*U*^12^	*U*^13^	*U*^23^
Sn1	0.0318 (3)	0.0405 (3)	0.0336 (3)	−0.00191 (17)	0.0148 (2)	0.00056 (18)
O1	0.033 (2)	0.057 (3)	0.031 (2)	−0.0018 (16)	0.0131 (19)	0.0003 (18)
O2	0.034 (2)	0.055 (3)	0.040 (3)	−0.0061 (17)	0.014 (2)	−0.0040 (18)
O3	0.039 (3)	0.084 (3)	0.047 (3)	0.001 (2)	0.019 (2)	0.004 (2)
C7	0.038 (4)	0.044 (4)	0.051 (4)	−0.003 (3)	0.022 (3)	0.003 (3)
C14	0.034 (3)	0.036 (3)	0.036 (4)	0.002 (2)	0.014 (3)	0.005 (3)
C8	0.033 (3)	0.041 (3)	0.040 (4)	−0.001 (2)	0.012 (3)	0.000 (3)
C1	0.034 (3)	0.049 (3)	0.063 (5)	−0.003 (3)	0.027 (3)	0.004 (3)
O4	0.033 (2)	0.066 (3)	0.038 (3)	−0.0080 (18)	0.016 (2)	−0.0043 (19)
C9	0.042 (4)	0.068 (4)	0.040 (4)	−0.001 (3)	0.015 (3)	−0.003 (3)
C6	0.040 (4)	0.064 (4)	0.045 (4)	−0.003 (3)	0.017 (3)	0.001 (3)
C13	0.047 (4)	0.079 (5)	0.046 (4)	−0.006 (4)	0.018 (4)	−0.001 (4)
C16	0.074 (4)	0.036 (3)	0.065 (4)	0.006 (3)	0.044 (4)	0.004 (3)
C2	0.034 (3)	0.077 (5)	0.063 (5)	−0.010 (3)	0.021 (3)	−0.005 (4)
C10	0.038 (4)	0.097 (6)	0.062 (5)	−0.008 (4)	0.023 (4)	−0.002 (4)
C5	0.069 (5)	0.077 (5)	0.067 (6)	0.005 (4)	0.045 (5)	0.004 (4)
C15	0.060 (4)	0.037 (3)	0.056 (4)	−0.007 (3)	0.032 (3)	0.004 (3)
C11	0.034 (4)	0.107 (7)	0.068 (6)	−0.017 (4)	0.011 (4)	−0.010 (5)
C3	0.045 (4)	0.091 (6)	0.091 (7)	−0.013 (4)	0.031 (5)	−0.007 (5)
C4	0.060 (5)	0.089 (6)	0.095 (7)	−0.008 (4)	0.052 (5)	0.010 (5)
C12	0.050 (4)	0.102 (6)	0.044 (5)	−0.012 (4)	0.001 (4)	−0.009 (4)
F1	0.082 (3)	0.166 (5)	0.074 (4)	0.000 (3)	0.053 (3)	−0.005 (3)
F3	0.049 (3)	0.196 (6)	0.092 (4)	−0.018 (3)	0.038 (3)	−0.011 (4)
F4	0.066 (3)	0.243 (7)	0.054 (3)	−0.034 (4)	−0.003 (3)	−0.030 (4)
F2	0.048 (3)	0.183 (5)	0.115 (4)	−0.034 (3)	0.031 (3)	−0.013 (4)

### Geometric parameters (Å, °)

**Table:**

Sn1—O1	2.534 (4)	C13—C12	1.364 (9)
Sn1—O1^i^	2.793 (4)	C13—H13	0.9300
Sn1—O2	2.163 (4)	C16—H16A	0.9600
Sn1—O3	2.424 (4)	C16—H16B	0.9600
Sn1—O4	2.155 (4)	C16—H16C	0.9600
Sn1—C15	2.093 (6)	C2—C3	1.359 (9)
Sn1—C16	2.088 (6)	C2—H2	0.9300
O1—C14	1.252 (7)	C10—C11	1.350 (10)
O2—C14	1.283 (7)	C10—F3	1.355 (7)
O3—C7	1.249 (8)	C5—C4	1.353 (11)
C7—O4	1.284 (8)	C5—F1	1.356 (9)
C7—C1	1.497 (8)	C15—H15A	0.9600
C14—C8	1.490 (8)	C15—H15B	0.9600
C8—C13	1.372 (9)	C15—H15C	0.9600
C8—C9	1.394 (8)	C11—C12	1.369 (10)
C1—C6	1.363 (9)	C11—H11	0.9300
C1—C2	1.385 (8)	C3—F2	1.355 (8)
C9—C10	1.360 (8)	C3—C4	1.357 (11)
C9—H9	0.9300	C4—H4	0.9300
C6—C5	1.377 (9)	C12—F4	1.344 (8)
C6—H6	0.9300		
			
C16—Sn1—C15	156.8 (3)	C12—C13—C8	118.3 (7)
C16—Sn1—O4	98.49 (19)	C12—C13—H13	120.8
C15—Sn1—O4	98.00 (18)	C8—C13—H13	120.8
C16—Sn1—O2	96.8 (2)	Sn1—C16—H16A	109.5
C15—Sn1—O2	100.32 (19)	Sn1—C16—H16B	109.5
O4—Sn1—O2	86.84 (16)	H16A—C16—H16B	109.5
C16—Sn1—O3	89.49 (19)	Sn1—C16—H16C	109.5
C15—Sn1—O3	85.97 (18)	H16A—C16—H16C	109.5
O4—Sn1—O3	56.72 (16)	H16B—C16—H16C	109.5
O2—Sn1—O3	143.56 (17)	C3—C2—C1	117.7 (7)
C16—Sn1—O1	89.44 (18)	C3—C2—H2	121.1
C15—Sn1—O1	87.67 (17)	C1—C2—H2	121.1
O4—Sn1—O1	141.79 (15)	C11—C10—F3	118.0 (6)
O2—Sn1—O1	55.04 (15)	C11—C10—C9	123.5 (7)
O3—Sn1—O1	161.28 (16)	F3—C10—C9	118.5 (7)
C14—O1—Sn1	84.2 (3)	C4—C5—F1	118.5 (7)
C14—O2—Sn1	100.6 (4)	C4—C5—C6	122.0 (8)
C7—O3—Sn1	86.2 (4)	F1—C5—C6	119.5 (7)
O3—C7—O4	119.4 (6)	Sn1—C15—H15A	109.5
O3—C7—C1	121.8 (6)	Sn1—C15—H15B	109.5
O4—C7—C1	118.8 (6)	H15A—C15—H15B	109.5
O1—C14—O2	120.0 (5)	Sn1—C15—H15C	109.5
O1—C14—C8	121.2 (5)	H15A—C15—H15C	109.5
O2—C14—C8	118.8 (5)	H15B—C15—H15C	109.5
C13—C8—C9	120.4 (6)	C10—C11—C12	117.1 (7)
C13—C8—C14	120.4 (6)	C10—C11—H11	121.5
C9—C8—C14	119.1 (6)	C12—C11—H11	121.5
C6—C1—C2	121.0 (6)	F2—C3—C4	119.0 (7)
C6—C1—C7	120.2 (6)	F2—C3—C2	118.0 (8)
C2—C1—C7	118.9 (6)	C4—C3—C2	122.9 (7)
C7—O4—Sn1	97.6 (4)	C5—C4—C3	118.0 (7)
C10—C9—C8	117.9 (6)	C5—C4—H4	121.0
C10—C9—H9	121.1	C3—C4—H4	121.0
C8—C9—H9	121.1	F4—C12—C13	119.2 (7)
C1—C6—C5	118.4 (7)	F4—C12—C11	118.0 (7)
C1—C6—H6	120.8	C13—C12—C11	122.8 (7)
C5—C6—H6	120.8		
			
C16—Sn1—O1—C14	−96.4 (3)	C1—C7—O4—Sn1	−174.4 (4)
C15—Sn1—O1—C14	106.7 (3)	C16—Sn1—O4—C7	−85.4 (4)
O4—Sn1—O1—C14	6.7 (4)	C15—Sn1—O4—C7	78.2 (3)
O2—Sn1—O1—C14	2.4 (3)	O2—Sn1—O4—C7	178.1 (3)
O3—Sn1—O1—C14	176.9 (4)	O3—Sn1—O4—C7	−1.6 (3)
C16—Sn1—O2—C14	82.1 (3)	O1—Sn1—O4—C7	174.6 (3)
C15—Sn1—O2—C14	−82.1 (3)	C13—C8—C9—C10	0.9 (10)
O4—Sn1—O2—C14	−179.7 (3)	C14—C8—C9—C10	−176.7 (6)
O3—Sn1—O2—C14	−179.4 (3)	C2—C1—C6—C5	0.5 (10)
O1—Sn1—O2—C14	−2.4 (3)	C7—C1—C6—C5	−179.4 (6)
C16—Sn1—O3—C7	102.2 (4)	C9—C8—C13—C12	−1.1 (10)
C15—Sn1—O3—C7	−100.6 (4)	C14—C8—C13—C12	176.4 (6)
O4—Sn1—O3—C7	1.7 (3)	C6—C1—C2—C3	0.0 (10)
O2—Sn1—O3—C7	1.3 (5)	C7—C1—C2—C3	179.9 (6)
O1—Sn1—O3—C7	−171.1 (4)	C8—C9—C10—C11	0.2 (12)
Sn1—O3—C7—O4	−2.7 (5)	C8—C9—C10—F3	178.8 (6)
Sn1—O3—C7—C1	174.7 (5)	C1—C6—C5—C4	−1.4 (11)
Sn1—O1—C14—O2	−3.8 (4)	C1—C6—C5—F1	179.5 (6)
Sn1—O1—C14—C8	173.0 (4)	F3—C10—C11—C12	−179.6 (7)
Sn1—O2—C14—O1	4.5 (5)	C9—C10—C11—C12	−0.9 (13)
Sn1—O2—C14—C8	−172.3 (4)	C1—C2—C3—F2	177.8 (6)
O1—C14—C8—C13	−177.3 (6)	C1—C2—C3—C4	0.5 (12)
O2—C14—C8—C13	−0.5 (8)	F1—C5—C4—C3	−179.1 (7)
O1—C14—C8—C9	0.2 (8)	C6—C5—C4—C3	1.8 (12)
O2—C14—C8—C9	177.0 (5)	F2—C3—C4—C5	−178.7 (7)
O3—C7—C1—C6	170.9 (6)	C2—C3—C4—C5	−1.3 (13)
O4—C7—C1—C6	−11.6 (8)	C8—C13—C12—F4	−177.3 (7)
O3—C7—C1—C2	−9.0 (9)	C8—C13—C12—C11	0.4 (12)
O4—C7—C1—C2	168.4 (5)	C10—C11—C12—F4	178.3 (8)
O3—C7—O4—Sn1	3.1 (6)	C10—C11—C12—C13	0.6 (13)

Symmetry codes: (i) −*x*, *y*, −*z*+1/2.

### Hydrogen-bond geometry (Å, °)

**Table:**

*D*—H···*A*	*D*—H	H···*A*	*D*···*A*	*D*—H···*A*
C11—H11···F4^ii^	0.93	2.49	3.326 (8)	149
C15—H15B···O4^iii^	0.96	2.52	3.474 (8)	171

Symmetry codes: (ii) −*x*−1, *y*, −*z*−1/2; (iii) −*x*, −*y*+1, −*z*.
